# High‐throughput 3D modelling to dissect the genetic control of leaf elongation in barley (*Hordeum vulgare*)

**DOI:** 10.1111/tpj.14225

**Published:** 2019-02-22

**Authors:** Ben Ward, Chris Brien, Helena Oakey, Allison Pearson, Sónia Negrão, Rhiannon K. Schilling, Julian Taylor, David Jarvis, Andy Timmins, Stuart J. Roy, Mark Tester, Bettina Berger, Anton van den Hengel

**Affiliations:** ^1^ Australian Center for Visual Technologies University of Adelaide Adelaide SA 5005 Australia; ^2^ Australian Plant Phenomics Facility The Plant Accelerator^®^ School of Agriculture Food & Wine University of Adelaide Urrbrae SA 5064 Australia; ^3^ School of Agriculture Food & Wine and Waite Research Institute University of Adelaide Urrbrae SA 5064 Australia; ^4^ Phenomics and Bioinformatics Research Centre School of Information Technology and Mathematical Sciences University of South Australia Adelaide 5001 Australia; ^5^ ARC Centre of Excellence in Plant Energy Biology The University of Adelaide PMB 1, Glen Osmond Adelaide South Australia 5064 Australia; ^6^ Australian Centre for Plant Functional Genomics PMB 1, Glen Osmond Adelaide South Australia 5064 Australia; ^7^ Division of Biological and Environmental Sciences and Engineering (BESE) King Abdullah University of Science and Technology (KAUST) Thuwal 23955‐6900 Saudi Arabia

**Keywords:** 3D modelling, phenotyping, leaf elongation, cereals, shoot architecture, quantitative trait locus (QTL), salinity, technical advance

## Abstract

To optimize shoot growth and structure of cereals, we need to understand the genetic components controlling initiation and elongation. While measuring total shoot growth at high throughput using 2D imaging has progressed, recovering the 3D shoot structure of small grain cereals at a large scale is still challenging. Here, we present a method for measuring defined individual leaves of cereals, such as wheat and barley, using few images. Plant shoot modelling over time was used to measure the initiation and elongation of leaves in a bi‐parental barley mapping population under low and high soil salinity. We detected quantitative trait loci (QTL) related to shoot growth *per se*, using both simple 2D total shoot measurements and our approach of measuring individual leaves. In addition, we detected QTL specific to leaf elongation and not to total shoot size. Of particular importance was the detection of a QTL on chromosome 3H specific to the early responses of leaf elongation to salt stress, a locus that could not be detected without the computer vision tools developed in this study.

## Significance Statement

To dissect overall shoot growth in small grain cereals, a computer vision‐based approach was developed for the measurement of individual leaves, which requires only few input images, making image acquisition fast and high throughput. The applicability of this approach is shown by mapping QTL for leaf elongation in a barley mapping population in response to salinity.

## Introduction

Small grain cereals are the staple food for the majority of the world's population, with a combined harvest of 1.6 billion tons in 2016 for rice, wheat and barley alone (http://www.fao.org/faostat/). To ensure sufficient cereal production for a growing population, the current rate of yield improvement in cereals has to grow by more than 35% (Tester and Langridge, 2010). Modern cultivars will need to combine superior stress tolerance with a high yield potential. Gains in yield stability and yield potential in the future will rely on improved resource use efficiency and optimized shoot growth (Sheehy *et al*., 2001; Parry *et al*., 2011; Reynolds *et al*., 2011; Sheehy and Mitchell, 2015). Improving shoot growth and shoot structure to, for example, improve light interception and canopy structure requires an understanding of the physiological processes involved, the influence of the environment and the underlying genetic control. Large‐scale studies of shoot growth are thus essential.

Recent advances in plant phenotyping technology now make it possible to measure shoot growth at high throughput, thus enabling forward genetic screens. However, most high‐throughput approaches so far used 2D imaging and treated the plant shoot as a single object (Campbell *et al*., 2015, 2017; Al‐Tamimi *et al*., 2016; Feldman *et al*., 2017; Muraya *et al*., 2017). Studies analyzing shoot morphology in detail and at high throughput have focused on large plants with a fairly simple architecture, like maize (Cabrera‐Bosquet *et al*., 2016; Santos and Rodrigues, 2016; Zhang *et al*., 2017) or young dicotyledons (Golbach *et al*., 2016; Scharr *et al*., 2016; Liu *et al*., 2017). Recovering the shoot morphology of small grain cereals, however, is a lot more challenging, due to their thin, flexible leaves that often twist along their longitudinal axis and have an irregular 3D shoot structure.

Previous studies modelling the 3D structure of plants, such as wheat and rice, have relied on either sophisticated imaging instruments such as LIDAR (Dornbusch *et al*., 2012; Paulus *et al*., 2014; Vadez *et al*., 2015; McCormick *et al*., 2016), or the acquisition of up to hundreds of images (Frolov *et al*., 2016; Nguyen *et al*., 2016; Pound *et al*., 2016) and, to our knowledge, none of these approaches has been employed at high throughput over time. Silhouette carving is a common approach, generating a model by finding the intersection of the back‐projections of the silhouette of the plant in each view (Gibbs *et al*., 2017), in some cases followed by segmenting that model into individual leaves. Silhouette carving often needs hundreds of images, especially for complex structures. As a result, image acquisition can be time consuming (Frolov *et al*., 2016; Nguyen *et al*., 2016) and thus becomes a limiting factor. Our approach is significantly different from techniques used thus far because it requires only a few images (five in this study) and is therefore feasible at high throughput with short imaging times, while processing can run during non‐imaging periods. Using a small number of images, our approach generates a large number of potential 3D leaf models that are plausible given the acquired set of images before selecting the most likely model with the fewest number of leaves.

To demonstrate the applicability of our approach for high‐throughput research, we performed structural analyses of barley shoots of a mapping population to measure leaf initiation and elongation over time. Moreover, we studied the genetic mechanisms underlying the effects of salt stress on shoot growth and leaf elongation. We were able to detect QTL common to both shoot growth using 2D imaging and leaf elongation using structural analysis, validating our approach. More importantly, we detected novel QTL and candidate genes for leaf specific traits. Shoot growth of small grain cereals can now be dissected into its components to identify underlying genetic mechanisms of growth of individual leaves, and the maintenance of their growth under salt stress. Our technique could have a significant impact in the understanding of shoot growth and structure, its genetic control, and how it is influenced by different environmental stresses.

## Results

### Leaf tracking can be achieved with a small number of images

Our method was designed for the 3D reconstruction of plant shoots from multiple images. Due to the thin, featureless leaves of cereal shoots, and their relatively uniform colour, accurate reconstruction is infeasible using standard feature matching techniques. Hence, it is common to apply silhouette carving approaches (Gibbs *et al*., 2017), which only require identification of the pixels belonging to the plant in each image. However, as there are many 3D objects which will produce the same silhouette in an image, these approaches can require a large number of images to determine the correct structure. If too few images are available, the results of silhouette carving will include spurious structures not present in the actual plant.

In this study, we demonstrate that from a small set of images it is possible to reconstruct and track shoot structure using only the silhouette of the plant in each view, by applying prior knowledge of plant structure to determine the most likely structure from the possible 3D shapes that match the silhouettes. To find the most likely 3D plant model given the five input images, we used a generate‐and‐test method which generated plausible 3D plant models and evaluated them against the image set to determine the optimal reconstruction.

The process followed five key steps outlined below and detailed in Experimental Procedures.

1. Camera Calibration: Recovering the 3D shape of an object (e.g. leaf) visible in multiple images requires camera calibration to determine the position, orientation, and intrinsic parameters of the cameras which captured the images. Camera calibration was achieved with a calibration object with chess board patterns at 90‐degree angles captured in all camera views (Figure S1). Alternatively, smaller targets that display easily recognized patterns at a variety of orientations can be used. (Figure S1).
2. Extracting 2D structure: The goal of the reconstruction process was to obtain a 3D path describing the axis of each leaf. This allowed for measurements of leaf length and angle. A set of 2D paths extracted from the silhouette for each view were used as estimates of the projection of the set of 3D leaf axes. First, pixels belonging to the plant were identified using a classifier (Figure 1a,b), before identifying axis points on the leaves and the 2D width and orientation of the leaves at these points. These axis points were then connected to form a set of 2D paths covering each leaf in each image (Figure 1c).

**Figure 1:**
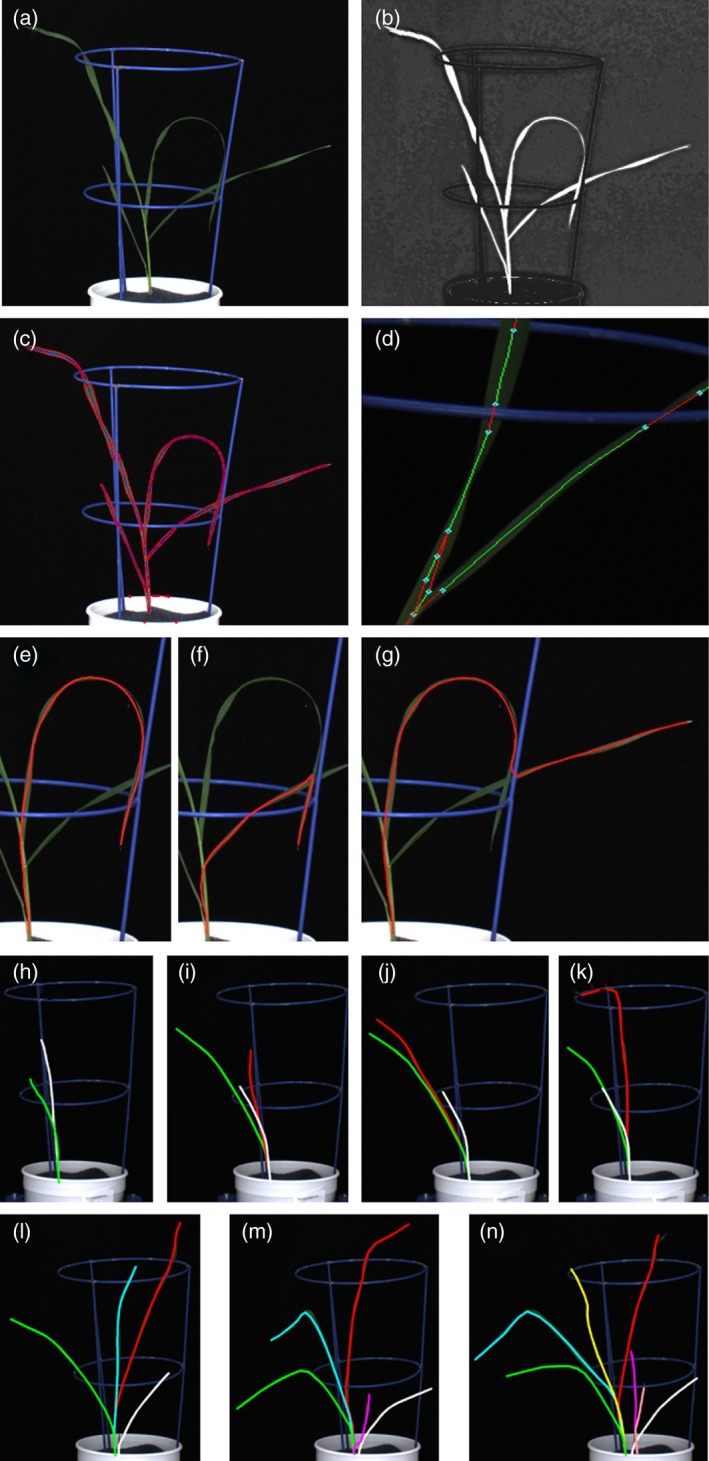
Image processing steps to generate 3D shoot models and track leaves over time. (a) Example RGB input image and (b) resulting image after pixel classification with white being identified as plant. (c) 2D segments fitted to leaves to generate (d) 2D paths along leaf axes, connecting disconnect points. (e) Path between end points and basepoints were established to generate individual leaf models. (f, g) Impossible leaf models, contradicting typical leaf angles and gravity were rejected. (h–n) Using 3D models of the same plants on subsequent imaging dates, individual leaves were labelled and traced over time.
3. Extracting 3D structure: We identified possible matching 2D segments in images from different views and used triangulation to determine the 3D position of points along the matched segments. This generated a set of 3D segments which were then connected in a graph structure (Figure 1d). By randomly traversing this graph, we generated a set of possible 3D leaf models (Figure 1e). Impossible leaf models were rejected, such as models consisting of a descending segment flanked by ascending segments either side (Figure 1f,g).
4. Plant reconstruction: Using the plausible individual leaf models, we generated a large number of possible plant models (100 000). We then evaluated these plant models and selected the best model based on the overlap with the silhouettes extracted from the input images, the number of leaves used, and the total leaf curvature.
5. Tracking leaf growth: We then compared leaf position, length, shape and growth between 3D models of the same plant on consecutive dates. This allowed identification of individual leaves (first, second leaf, etc.) as they emerged and enabled tracking of leaves over time (Figure 1h–n). At this stage, leaves can be identified by their order of appearance. The input images, however, are not sufficient to identify whether leaves belong to the main tiller or secondary tillers.

### Recovering shoot structure of barley provides novel traits for dissection of growth

To validate the image analysis proposed here for recovering shoot structure, we compared digital and manual measurements of total and individual leaf length (Figure S2), after manually determining the identity of each leaf in the digital results. For both wheat and barley, we get a high correlation between the manual and digital measurements of leaf length (*R* ^2^ between 0.97 and 0.99). The digital measurements tend to slightly underestimate leaf length, which is an issue of occlusion by the pot where the location of the shoot base has to be estimated. Overall, the method presented here provides high quality estimates of leaf length for thin‐leaved cereals (Figures S2 and S3) and is suitable for measuring shoot growth at the leaf level.

**Figure 2:**
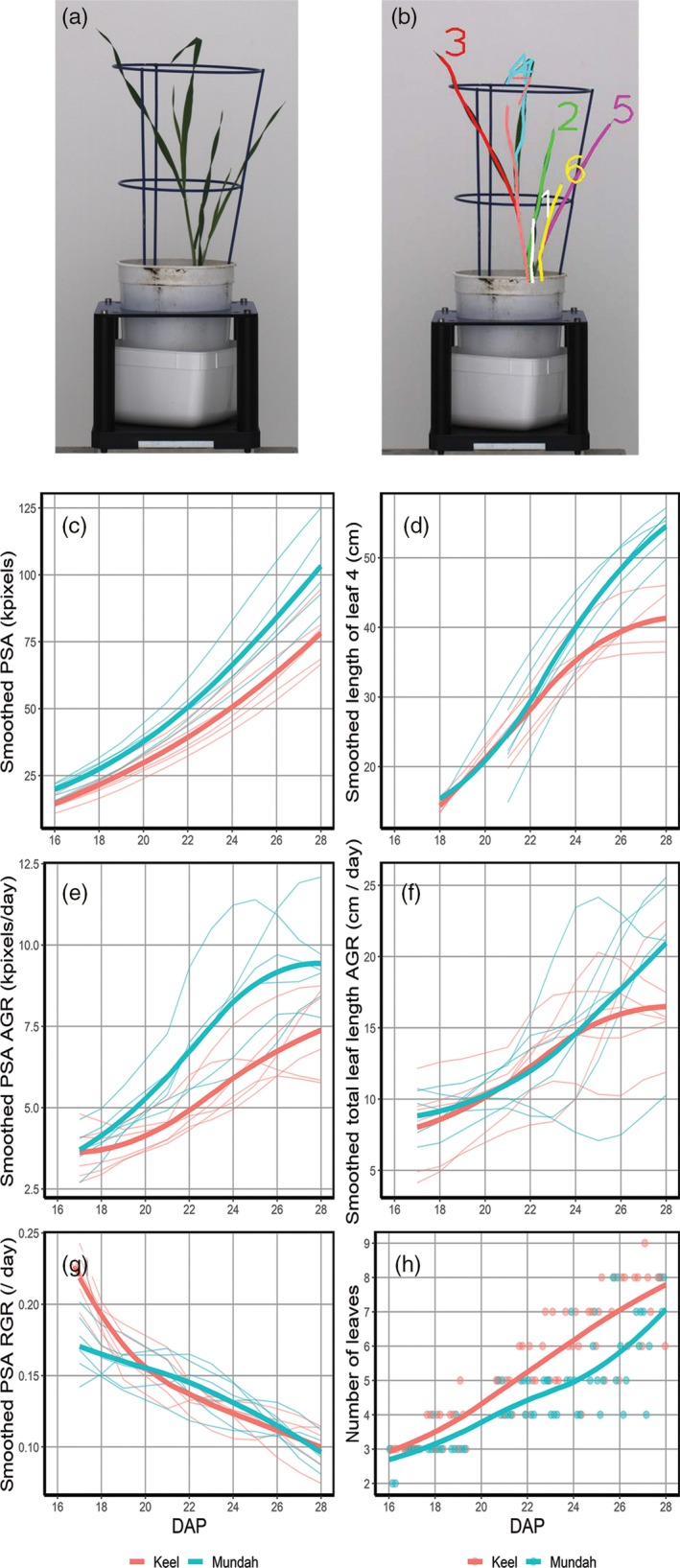
Comparison of traits extracted from 2D imaging and 3D modelling for Mundah and Keel barley (*Hordeum vulgare*) plants. (a) Original RGB image of an example plant next to the (b) corresponding 3D model with individually labelled leaves. Traits extracted from 2D imaging include smoothed projected shoot area (PSA; c), smoothed absolute growth rate (AGR, e) and smoothed relative growth rates (RGR, g). 3D modelling and leaf tracking enables (d) measurement of individual leaf length, (f) smoothed total length of leaves per plant and (h) number of leaves. Individual plants are depicted in thin lines, thicker lines are loess smooths representing the average trends for Mundah (blue) and Keel (red).

To compare the shoot growth of parents of a bi‐parental barley mapping population, Mundah and Keel, we fitted smoothing splines to overall shoot area (projected shoot area, PSA), total leaf length and individual leaf lengths over time. We then derived absolute growth rate (AGR) and the relative growth rate (RGR) from the smoothed curves (Figure 2c,e,g). Overall, Mundah showed a more prolific shoot growth compared with Keel as evident by the larger overall PSA and PSA AGR during the imaging period (Figure 2c,e). While Keel appeared to have a larger PSA RGR up until 20 days after planting (DAP), the RGR of Mundah was slightly higher than that of Keel for the period between 20 and 27 DAP (Figure 2g), ultimately leading to a larger final shoot size. Next, we analyzed the shoot structure in more detail using our approach, to gain insights into the traits responsible for the observed differences in shoot growth between the cultivars. For example, when analyzing the length of leaf 4, Mundah had a longer leaf 4 compared with Keel (Figure 2d). The AGR for total leaf length was also increased for Mundah compared with Keel, this increase became more evident towards the later growth stages, from 24 DAP (Figure 2f). Regarding PSA AGR, this trait differed between the two cultivars already at 18 DAP (Figure 2e). This suggests that leaf length is not the only determinant of shoot size, but other factors, such as leaf width may also be important. Leaf number is another key driver for shoot size, and Keel had more leaves compared with Mundah (Figure 2h). Therefore, the smaller shoot size of Keel is likely to be due to shorter leaves, and potentially smaller leaf width, not less leaves. The phenotypic differences between the cultivars in shoot growth and its component traits suggested the presence of genetic differences, which we set out to explore further.

### Effects of soil salinity on leaf elongation and initiation over time

**Figure 3:**
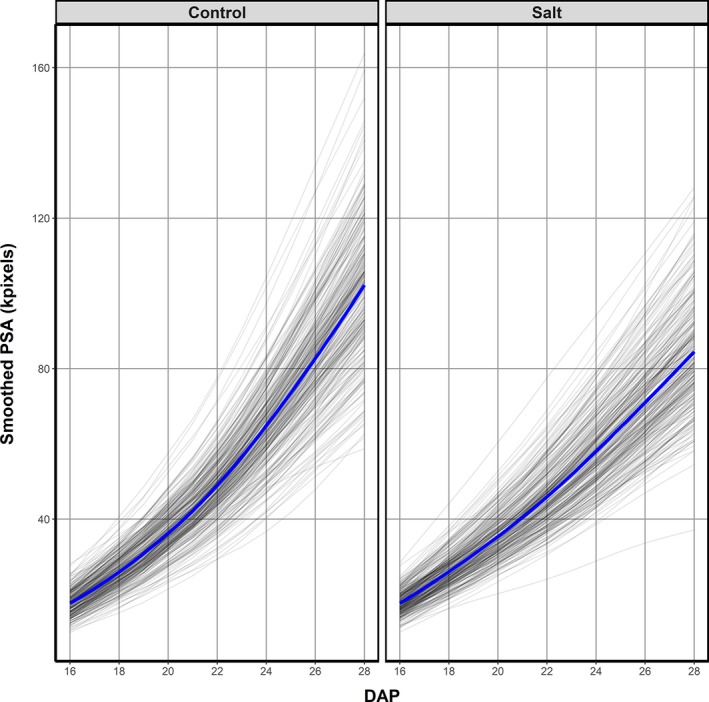
Effect of 200 mm NaCl in soil solution on shoot growth in the Mundah × Keel barley (*Hordeum vulgare*) mapping population. The trends in the smoothed projected shoot area (PSA) for control and salt‐treated individual plants of the Mundah **×** Keel mapping population during the period of imaging. The blue lines are the loess smooths of the trends for the individual plants and represent the average trends for the control and salt‐treated plants. Salt application occurred at 20 days after planting (DAP).

We investigated the power of our approach to measure shoot growth responses under stress conditions. As soil salinity is known to have an immediate effect on shoot growth (Munns and Tester, 2008), the aim was to dissect shoot growth reduction under salt stress into the components of leaf initiation and leaf elongation. To achieve this, a bi‐parental mapping population of recombinant inbred lines (RIL) from a Mundah × Keel cross was grown under low (control; 0 mm NaCl) and high soil salinities (salt; 200 mm NaCl). Using PSA as a measure for total shoot size, the effect of salinity on plant growth became evident by a reduced PSA in salt compared with control conditions less than a week after salt application (Figure 3). At the completion of imaging, mean PSA of the salt‐stressed plants was reduced by approximately 15% compared with control plants, and the highest salt‐stressed PSA values were approximately 25% lower than those for the largest control plants.

**Figure 4:**
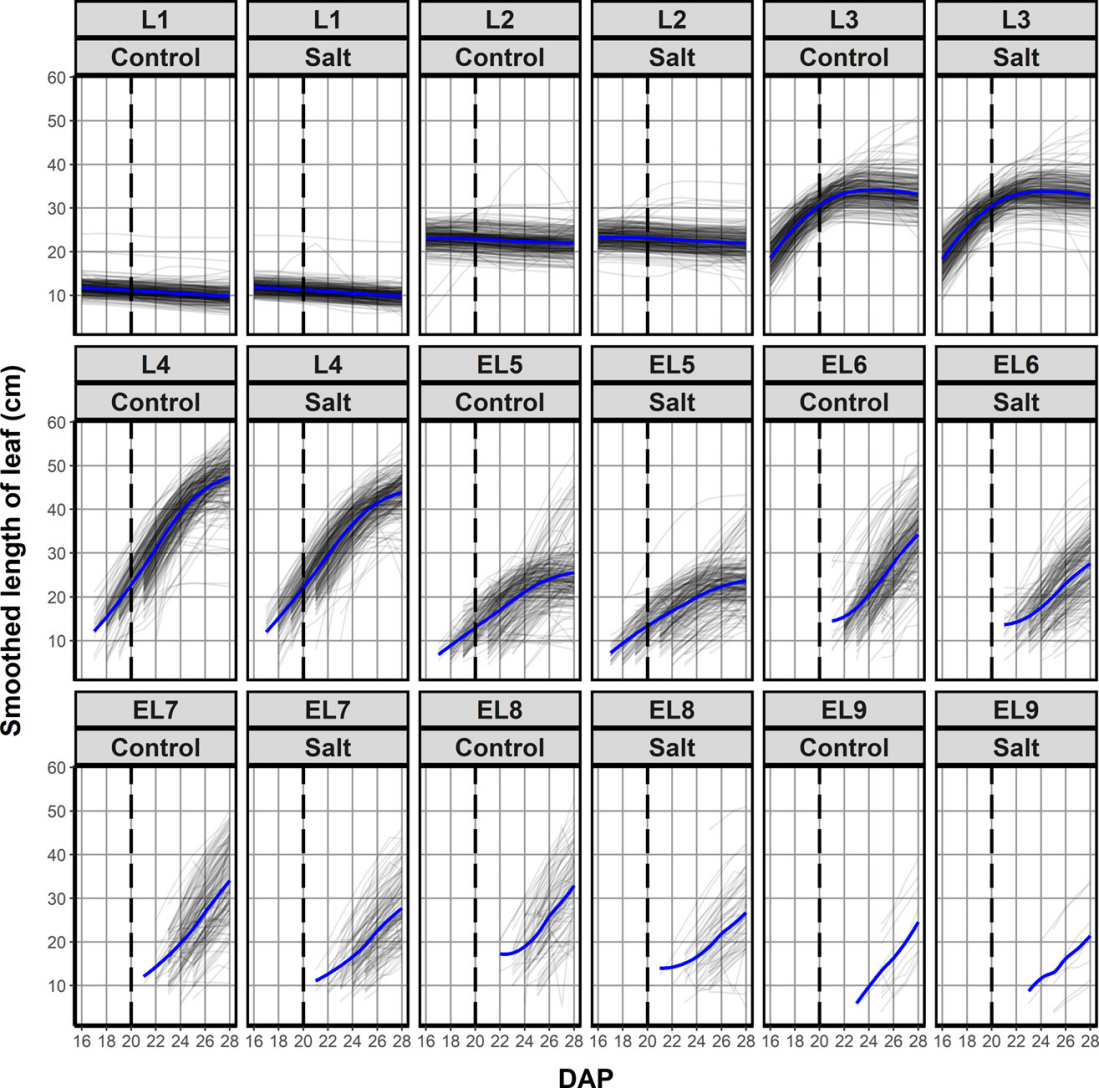
Effect of 200 mm NaCl in soil solution on individual leaf initiation and leaf elongation in the Mundah × Keel barley (*Hordeum vulgare*) mapping population. Smoothed length of individual leaves for each plant are depicted over time for control and salt‐treated plants. Leaves 1–4 (L1–L4) were leaves on the main tiller, leaves emerging after leaf 4 could be on a side tiller or main tiller and are thus labelled as emerging leaf (EL) for differentiation. The fifth leaf to emerge (emerging leaf 5; EL5) in the majority of plants was the first leaf of tiller 1. For the majority of plants, EL6 corresponded to the second leaf of the first side tiller, EL7 to leaf 5 on the main tiller and EL8 to the first leaf of the second side tiller, respectively. The blue lines are the loess smooths of the trends for the individual plants and represent the average trends for the control and salt‐treated plants. Salt application occurred at 20 days after planting (DAP).

To test the feasibility of applying our approach to recover plant structure at high throughput, we extracted structural information for the lines of the Mundah × Keel RIL population under control and salt stress. We were able to monitor individual leaf development, and measure leaf elongation during the imaging period from 16 to 28 DAP (Figure 4). At the start of imaging, leaves 1 and 2 were fully expanded, while leaf 3 was still growing, reaching a plateau at about 23 DAP. Salt stress was applied to one individual of a pair of the same genotype at 20 DAP, when leaf 4 had emerged in most plants. As expected, the mean length of leaf 4 was smaller in salt‐stressed plants compared with the control (43.9 cm – salt, 47.3 cm – control), an effect also seen for later developing leaves. The fifth leaf to emerge (emerging leaf 5; EL5) in the majority of plants was the first leaf of tiller 1, not leaf 5 on the main tiller, and therefore explaining the shorter overall size of EL5 compared with leaf 4. The mean length of EL5 was apparently less affected by high salinity than the length of subsequent leaves, possibly due to its overall shorter length. The following leaves to emerge (EL6, EL7 and EL8), however, displayed a clear reduction in mean length under salt compared with control conditions. In addition to leaf elongation, we also observed that leaf initiation was affected by salt stress. A higher number of plants had eight or more leaves initiated under control conditions compared with salt‐treated plants. Overall, our approach provided new metrics of previously uncharacterized traits, leaf elongation and initiation, which can be further used to identify the genetic mechanisms controlling shoot growth and structure under control and stress conditions.

### QTL mapping reveals overlap and distinction between genetic control of whole‐shoot growth, leaf elongation and leaf initiation

To dissect the genetic basis of shoot growth, we fitted smoothing splines to PSA, total leaf length and individual leaf lengths over time. We then calculated smoothed values for PSA (PSA.smooth) in control and salt for the time after salt application. In addition, we calculated daily AGR and RGR for PSA and total and mean leaf length (total.length.smooth, mean.length.smooth) from 21 to 25 DAP, as well as AGR and RGR over each of the intervals 21–24 and 24–28 DAP (Table S1). Number of leaves (num.leaves) and leaf 4 length (L4.length.smooth) were tested for individual days only (Table S1). These derived traits maintained data at the individual plant level and were used in subsequent analyses. All traits that showed sufficient genetic variation evidenced by a heritability greater than 0.1 (Table S2) underwent QTL analysis.

In total, we detected over 60 significant QTL across all seven chromosomes (Tables S3–S5), with some QTL located directly adjacent to each other, most likely representing the same locus. The low limit of detection (LOD) scores for some QTL are reflective of the whole genome average interval mapping (WGAIM) method to detect and quantify smaller effect QTL for population sizes such as the one used here. We observed that the number of QTL detected differed greatly between the traits analyzed. Most strikingly, when using PSA.smooth as an input trait, only a single QTL on chromosome 2H was detected. By contrast, numerous QTL were detected when using PSA.smooth.AGR or PSA.smooth.RGR (Table S3). Several of the QTL for PSA.smooth.AGR and PSA.smooth.RGR had higher LOD scores than the PSA.smooth QTL and also detected novel candidate genes, highlighting the importance of applying growth models. While some QTL were stable over time, many QTL were transient and only appeared at individual time points. In addition, we detected a greater number of QTL under salinity compared with control conditions (47 salt, 18 control). Most salt related QTL for PSA.smooth.AGR and PSA.smooth.RGR were found at 24 DAP, while the majority of QTL for L4.length.smooth appeared earlier, at 22 and 23 DAP, with the two largest effect QTL observed at 22 DAP (Table S3).

**Figure 5:**
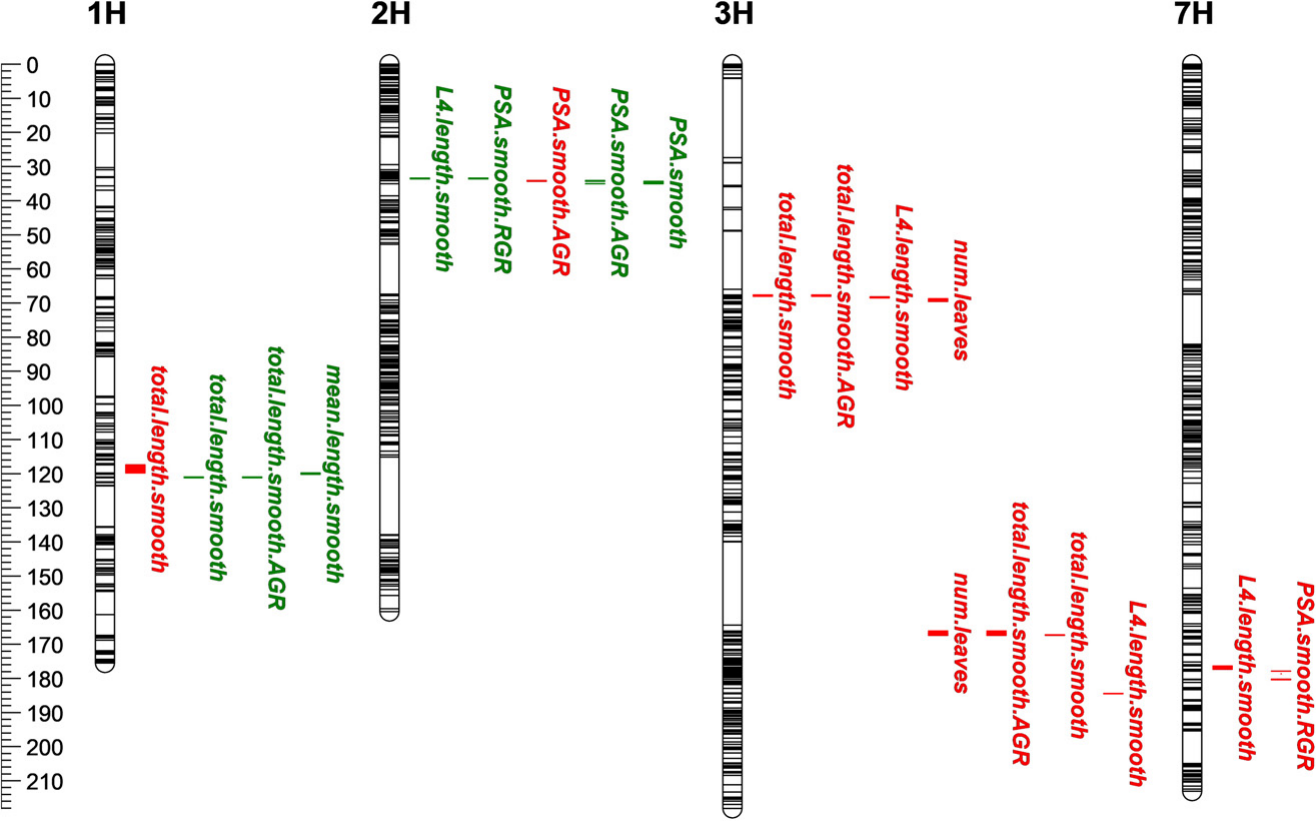
QTL map of 2D and 3D imaging traits highlighting selected QTL mapped in the Mundah × Keel barley (*Hordeum vulgare*) mapping population. QTL are indicated with left and right borders. QTL indicated in green were detected under control conditions, QTL indicated in red were detected in salinity. The *y*‐axis scale is in cM.

As mentioned above, QTL analysis for PSA.smooth only detected a single QTL. The QTL on the short arm of chromosome 2H at 34.25 cM for PSA.smooth at 24 and 28 DAP was also detected for PSA.smooth.AGR during the early time intervals between 21 and 24 DAP (Figure 5, Table S3) with a significant QTL for PSA.smooth.RGR in close proximity (33.43–33.62 cM), possibly representing the same locus. Overall, the genomic region appears to play an important role in the control of shoot growth *per se*, with LOD scores between 3.4 and 6.7 and the Mundah allele having a positive effect on shoot growth. Analysis of the annotated genes underlying the genetic region of the QTL at 34.25 cM, found the well known developmental gene pseudo‐response regulator Photoperiod‐H1 (*Ppd‐H1*) (Table S5) which was confirmed to be segregating within the Mundah × Keel RIL population by using a *Ppd‐H1* specific marker. A QTL for L4.length.smooth under control at 21 DAP was also mapped to 33.43 cM on chromosome 2H, however, this QTL had a negative effect, with the allele for increased leaf 4 length coming from Keel. There were several QTL at other locations which had negative effects for PSA.smooth.RGR. On chromosome 7H, between 177.93 cM and 180.42 cM, we detected two adjacent QTL for PSA.smooth.RGR for 22–23, 23–24 and 21–24 DAP (Figure 5). Both QTL had high LOD scores (4.5 for 177.93–180.24 cM, 6.3 for 180.24 –180.42 cM), each accounting for approximately 5% of the genetic variation (Table S3). Several transcription factors and an auxin‐related gene were found underlying this genetic region (Table S5), but require further fine mapping.

When analyzing the growth patterns of the parents Mundah and Keel, we observed similarities but also differences between the AGR curves for PSA and total leaf length, which could reveal differences in their genetic control. When examining QTL for leaf length specific traits, we found a QTL co‐located with another well known developmental gene, *FLOWERING LOCUS T*. The QTL on chromosome 1H for total.length.smooth (117.22–119.78 cM) under salt at 24 DAP shares its flanking marker with a QTL at 119.78 cM for mean.length.smooth under control conditions at 21 and 24 DAP (119.78–120.21 cM) (Figure 5, Table S5). This QTL was only detected when analyzing leaf length specific traits, but not for any of the PSA‐related traits (Table S4). On chromosome 3H, we also detected a QTL region specific to salinity response for leaf length‐related traits. Four traits, total.length.smooth, total.length.smooth.AGR, L4.length.smooth and num.leaves, were mapped between 67.56 and 69.50 cM (Figure 5, Table S3). For all traits, the positive allele was from Mundah. This chromosomal region contains several dozen annotated genes (Table S5), with no obvious candidate gene.

The second salinity‐specific QTL region on chromosome 3H was found between 166.09 and 167.40 cM (Figure 5), with significant LOD scores for num.leaves, total.length.smooth and total.length.smooth.AGR at multiple time points (Table S3). Three annotated genes were found in this region (Table S5), with a potassium transporter family protein a possible candidate for the observed differences in salinity tolerance. In addition to the QTL for both leaf length traits and leaf number, there were also QTL specific to either leaf length or leaf number, such as QTL for total.length.smooth on chromosome 2HL at 9.13 cM as well as QTL for num.leaves on chromosome 5H between 51.83 and 56.80 cM (Tables S3 and S4).

When analyzing QTL specific to the length of leaf 4, the leaf emerging as salt was applied, the QTL with the highest LOD score (6.5) was found on chromosome 3H between 184.35 and 184.54 cM (Figure 5, Table S3). There was a positive effect from the Mundah allele, with an average increased leaf 4 length of 2.2 cm. Analyzing the latest release of the barley genome, we found six annotated genes between the flanking markers (Table S5). Two of the annotated genes (unknown function; ABC transporter G family member) have extremely low expression according to the Barley Genome Explorer (http://apex.ipk-gatersleben.de/apex), while expression of the cytochrome P450 superfamily protein is only detectable in grains, making these three genes unlikely candidates for a salt responsive leaf elongation QTL at vegetative growth. The remaining three candidates underlying this QTL included two expansin B2 genes and a glucan endo‐1,3‐beta‐glucosidase. All three genes were expressed in seedlings and members of these two protein families are known to be directly involved in cell expansion and growth. Since this QTL was only detected 2 days after salt application (Table S3), it is possible that this locus plays a role in the early adaptation to salinity stress, and thus making these candidate genes interesting targets for future studies into salt‐specific growth responses.

## Discussion

To further improve our knowledge of plant shoot growth, we need new high‐throughput methods to analyze and dissect shoot structure, enabling forward genetics studies. Unlike rigid and feature rich objects, plant shoots pose unique challenges, with nearly uniform colour, and thin, flexible leaves. Different approaches have been employed to address these issues and generate 3D models of plant shoots (Gibbs *et al*., 2017). Most focused on plants with simple structures, such as Arabidopsis, fairly rigid dicotyledons, or near‐symmetrical monocotyledons like maize (Liu *et al*., 2017; Muraya *et al*., 2017; Zhang *et al*., 2017). Only few studies have tackled the challenging task of reconstructing 3D models of wheat, barley or rice and have either focused on very young plants using skeletonisation (Cai and Miklavcic, 2012; Frolov *et al*., 2016) or used many dozens of images to produce a 3D volume model (Pound *et al*., 2014).

As a result of the varying approaches to 3D reconstruction, the resulting 3D models differ in the traits that can be extracted. In many cases, the result of 3D reconstruction is a volume or surface model of the shoot, describing the 3D space the plant occupies. These types of model are suitable to measure total leaf area, leaf angle distribution and leaf area distribution (Gibbs *et al*., 2017). However, they still treat the shoot as a single object without detailed information on individual leaves. Others dissect the 3D volume or surface model into sub‐components, such as leaf and stem, but this has mainly been focused on dicotyledons (Paproki *et al*., 2012; Golbach *et al*., 2016; Liu *et al*., 2017). Here, we take an approach, where we generate shoot models describing the 3D path of individual leaves in barley best describing the small number of input images. As a result, we can measure total leaf length, individual leaf length and leaf angle over time. The principles employed in this study also have the potential to be transferred to different species and plant types (see Experimental procedures).

We demonstrated the feasibility of our approach to recover the structure of small grain cereals by analyzing the shoot structure of hundreds of barley recombinant inbred lines (RILs). We investigated the effects of salinity on shoot growth and structure and confirmed that salinity not only impacts leaf elongation, but also leaf initiation, indicated by shorter and less leaves in the salt‐stressed plants (Figures 3 and 4). Although this type of response is known (Munns and Tester, 2008), the underlying genetics are not understood yet, primarily due to insufficient phenotyping methods.

Recent studies have shown the potential of using whole‐shoot phenotyping approaches for measuring growth and growth responses to environmental stresses, such as salinity, to elucidate the genetic architecture of shoot development (Campbell *et al*., 2015, 2017; Al‐Tamimi *et al*., 2016). In this study, two genetic regions linked to well characterized developmental regulators, *Ppd‐H1* and *FLOWERING LOCUS T*, were detected. *Ppd‐H1* and its role in barley development and leaf size has been described previously (Wang *et al*., 2010; Maurer *et al*., 2015; Digel *et al*., 2016; Neumann *et al*., 2017; Alqudah *et al*., 2018). Although the strongest LOD scores for *Ppd‐H1* were found under control conditions in our seedling‐stage experiment (Table S3) and other studies focused on leaf size and biomass *per se* (Digel *et al*., 2016; Neumann *et al*., 2017; Alqudah *et al*., 2018), the same locus has been reported to increase yield under salinity in a barley nested association mapping (NAM) population (Saade *et al*., 2016). While the *Ppd‐H1* locus was consistent over multiple time points, other QTL appeared to be more transient.

The transient nature of many QTL has been described in previous phenotyping experiments (Al‐Tamimi *et al*., 2016; Feldman *et al*., 2017; Muraya *et al*., 2017; Neumann *et al*., 2017), and highlights the importance of time course measurements. Analyzing the growth trajectories of rice during early seedling development and tillering allowed the dissection of the genetic control of these different developmental stages (Campbell *et al*., 2017). In addition, the ability to measure the growth response of rice immediately after salinity application enabled the identification of several loci linked to signaling genes, which were transient in nature (Al‐Tamimi *et al*., 2016). The appearance and disappearance of QTL observed in this study and others (Al‐Tamimi *et al*., 2016; Feldman *et al*., 2017; Muraya *et al*., 2017), may also explain why replication of QTL between studies is often difficult. The transient nature of QTL was highlighted here by the detection of a large effect QTL for length of leaf 4 under salinity (Figure 5; Table S3) only 2 days after salt application. The candidate genes for this QTL include expansins (Table S5), which are known to play a direct role in cell expansion (Cosgrove *et al*., 2002; Sampedro and Cosgrove, 2005), and could be responsible for the increased length of leaf 4 under salt in plants with the Mundah allele. Since the QTL was only found at a single time point early after salt application, it is likely to be part of the early adaptation to salinity stress, but more comprehensive studies would be required to confirm the role of the identified genes.

Besides the QTL associated with leaf length, we also detected QTL for the number of leaves. Multiple of those QTL overlapped with leaf length QTL, suggesting loci related to shoot growth *per se*. However, we also found QTL specific for leaf number (Table S4), suggesting that these loci may play a role in leaf initiation and could offer the potential of dissecting leaf initiation and leaf elongation from each other, offering the potential for manipulating shoot architecture.

As expected for complex traits like leaf growth, the genetic control for the traits we analyzed appeared to be multi‐genic with numerous QTL of small effect. Although the multi‐genic nature of shoot growth and structure poses a challenge in identifying suitable targets for crop improvement, the markers identified here have the potential to be used in a Genomic Selection approach, to predict the growth and salinity response of various marker combinations, as recently done in rice (Campbell *et al*., 2017). Overall, the 3D modelling approach presented here offers new opportunities to understand the complexity of shoot growth and shoot architecture in cereals.

## Experimental procedures

### Plant material, growth conditions and salt treatment

Comparisons of digital versus manual leaf counting and leaf length measurements were performed on 20 barley plants (*Hordeum vulgare* cv Hindmarsh) and 20 wheat plants (*Triticum aestivum* cv Kukri) planted at four time points between June 2017 and July 2017 at the Australian Plant Phenomics Facility, The Plant Accelerator^®^, South Australia (34°58′17.00″S, 138°38′23.00″). Plants were grown in free‐draining pots with 145 cm diameter and 190 cm height placed inside saucers. The potting medium consisted of a peat mix containing ready‐available nutrients and slow release fertilizer (Osmocote^®^ 16‐3‐9+te). Pots were maintained at field capacity during the growth period and digital imaging was performed as described below. Manual leaf counting and leaf length measurements were performed at eight time points in July 2017 on different aged plants to capture a range of developmental stages. Leaf length was measured manually from the leaf tip to the base of the plant, including both leaf blade and leaf sheath. Automated digital leaf length measurements (see below) were conducted on a subset of 10 wheat and 10 barley plants of varying size.

The F_2_:F_6_ barley RILs developed through single seed decent are from a cross between the two varieties Mundah (Yagan/O'Connor), which is early flowering and photoperiod insensitive, and Keel (CPI‐18197/Clipper//WI‐2645), which is early flowering, but photoperiod sensitive (Collins, 1998; Barr, 2000; Long *et al*., 2003). Despite this, both cultivars are monomorphic for the flowering locus *HvFT1*. A set of 216 RILs from the mapping population were used for a single controlled glasshouse phenotyping experiment along with both parents. Six replicate plants of both parents (Mundah and Keel) and partial replication of the Mundah × Keel RIL lines with 20% duplication were used for the experiment.

Growth experiments were conducted in the Smarthouse at the Australian Plant Phenomics Facility, The Plant Accelerator^®^, South Australia (34°58′17.00″S, 138°38′23.00″) between March and May 2015 as previously described with minor modifications to the soil moisture content (Takahashi *et al*., 2015; Tilbrook *et al*., 2017). Greenhouse temperatures were maintained on a 22°C day/15°C night cycle with no additional lighting. Three imbibed seeds were planted into pots with a 50% (v/v) University of California (UC) Davis, 35% (v/v) Cocopeat, 15% (v/v) clay/loam potting mix on benches at the back of the Smarthouse. Once seedlings reached two‐leaf stage, the seedlings were thinned to one evenly sized seedling per pot. At 15 DAP, seedlings were loaded onto the phenotyping system for automated image acquisition and watering. Once loaded onto the belt, pots were adjusted to a gravimetric water content of 17% (g/g) and seedlings continued to grow until 19 DAP. Salt application was performed by adding 220 ml of 0 or 340 mm NaCl to the saucer reaching a water content of 27% (g/g) immediately after salt treatment 20 DAP. Within several days, final salt concentrations of 0 and 200 mm NaCl were reached in the pots once water levels dropped back to 17% (g/g) through evapotranspiration and were maintained at that level until completion of the experiment 33 DAP.

### Experimental design

The experiment uses the 24 Lanes by 22 Positions in the SW Smarthouse of The Plant Accelerator^®^, Australian Plant Phenomics Facility, which is divided into six zones, each comprising four lanes by 22 positions. The design employed for the experiment is a split‐plot design in which two consecutive carts form a main plot. The main‐plot design is a nearly trend‐free, partially replicated design that assigns lines to main plots; the subplot design merely randomizes conditions (control, salt) to the two carts in each main plot. The design was generated using the R package DiGGer (Coombes, 2009).

### Phenotyping using RGB image capture and LemnaGrid 2D image analysis

To assess the data generated by the ‘conventional’ high‐throughput phenotyping systems, i.e. whole‐shoot image, data were obtained by the LemnaTec 3D Scanalyzer system (LemnaTec GmbH, Aachen, Germany) at The Plant Accelerator. Plants’ shoots were imaged daily, from 16 DAP until 33 DAP, in an imaging chamber using two 8‐megapixel‐visible/RGB cameras (Allied Vision Technologies, Germany, GT3300C– GigE). To determine the PSA, which has been shown to correlate with biomass (Golzarian et al., 2011; Hairmansis *et al*., 2014; Honsdorf *et al*., 2014; Campbell *et al*., 2015; Parent *et al*., 2015; Al‐Tamimi *et al*., 2016), the protocol from Al‐Tamimi *et al*. (Al‐Tamimi *et al*., 2016) was used with minor modifications: five side views (SV) at 0, 20, 40, 60 and 80° rotation and one top view (TV) were captured. PSA was estimated by the sum of area SV0 + area SV80 + area TV. A schematic of the data workflow from image capture to QTL analysis, including software used, are provided in Figure S4.

### Shoot 3D modelling and leaf tracking

#### Input data

For results in this manuscript, we used five side view images at 20 degree rotation from each other (see above), but the method is applicable to any number of rotated plant images or camera placement, provided two views are available for each leaf.

#### Calibration

A chess board pattern was printed on a 3D step structure and imaged with the side view cameras at the same angles as plants and the TV camera (Figure S1a,b). Camera intrinsic and extrinsic parameters were estimated using standard chessboard camera calibration techniques. Alternatively, smaller targets that display easily recognized patterns at a variety of orientations can be used (Figure S1).

#### Extracting 2D structure

To extract 2D paths describing the leaves, we first identified pixels belonging to the plant in each frame with a pixel classifier. Before classification, we applied mean‐shift filtering to the images to reduce noise while preserving edges. We then applied a linear Support Vector Machine (SVM) classifier trained on a set of manually labelled images, using colour and texture‐based features. The classifier output was treated as a probability of each pixel belonging to the plant (Figure 1b). A classifier was used instead of a simple thresholding approach to give robustness in cases where plant and background pixels can have similar appearance.

The features used for classification were pixel colour from the mean‐shift filtered image, and the output of a Difference of Gaussian filter at multiple scales, used to detect edges in the image. For training, a set of 10 manually labelled images were used, which are sufficient to represent the variation in leaf appearance over the set of plant images. OpenCV libraries were used for the classification, and for other image processing functions performed by the software. Small misclassified regions may appear in the classifier output where the colour and appearance of leaves are similar to the appearance of the pot or the background. Small background regions misclassified as belonging to the plant in one frame did not typically affect the reconstruction result, as the process uses multiple images for consensus. Likewise, the reconstruction process is able to compensate for gaps due to small regions of plant misclassified as background. A confusion matrix for the classifier output, tested on five images, is given in Figure S5, showing accurate identification of plant pixels.

To extract the 2D leaf structure from the classifier output, we located pixels corresponding to points on the axis of a leaf, and determined 2D leaf width and orientation at these points. To achieve this, we first convolved the classifier output images with shape masks corresponding to the expected appearance of leaf regions. Shape masks were applied across the range of possible orientations and scales, and pixels corresponding to local maxima in the convolution results were identified as axis points, with the scale and orientation of the mask which gave the strongest response at each point used to determine the corresponding leaf width and orientation (Figure 1c). Adjacent axis points were clustered and used to define 2D paths corresponding to segments of leaf axes in the images. Separate segments were joined across small gaps to cover leaf areas too thin to be identified by the pixel classifier (Figure 1c).

#### Extracting 3D structure

After identifying leaf segments in each frame, we then matched segments between frames, and triangulated corresponding axis points to determine their 3D position. For a set of points along each 2D segment, we performed a search along the corresponding epipolar lines in all other frames, and found any intersections between the epipolar lines and another 2D segment. All intersections with the same segment were grouped, and the point in the first frame and intersection point in the second frame were triangulated to determine the corresponding 3D point. The set of 3D points for a pair of 2D segments formed a 3D segment (Figure S1d). This set of extracted segments was filtered to remove overlapping points on segments (determined as points separated by <0.4 cm), and then to remove very small segments with a total length of <0.4 cm.

3D segments corresponded to partial sections of leaves. These segments were separated by gaps wherever part of a leaf was occluded in all views, by other leaves or by the carnation frame. To generate paths corresponding to complete leaves, we connected this set of segments in a graph structure, and searched the graph to find paths combining multiple segments, which could plausibly correspond to complete leaves. To construct the graph, possible graph edges connecting path end points and the nearest point on another path were sorted by ascending length, and graph edges were added in several stages. In the first stage, all edges with a length of <1.5 cm were added to the graph. In the second stage, edges covering larger gaps of up to 7.5 cm were added. This could potentially include a large number of edges. To limit the size of the graph, and therefore the processing time required to search the graph, edges were only added in this second stage if they met certain criteria. Edges were added if the angle between an edge and any edge already added for the same end point was >25°, if the majority of the length of the edge did not overlap with previously added edges, and if the majority of the length of the edge was within the image regions covered by the plant (Figure 1d).

Having generated this graph structure, we then identified nodes corresponding to possible end points of leaves, and nodes corresponding to possible base points (i.e. points at which the leaf meets the soil). End points were selected by taking the set of end points for the 2D segments extracted for each frame, and finding the node in the graph with projection into that frame closest to each 2D segment end point. Points in this end point set which were within the bounds of the pot were labelled as base points. If clusters of connected segments existed which do not include a base point, due to being separated from the main body of the plant by a large region of occlusion, the edge adding step of the graph‐building process was repeated, this time allowing connections covering larger gaps of up to 15 cm for nodes in a disconnected cluster.

Leaf models were then generated by finding paths through the graph from end points to base points. Starting from each end point, paths were generated with a random walk through the graph, and paths which reached a base point before reaching any other end point were retained. For each end point, path generation was attempted 10 000 times. B‐splines were fitted to these paths to smooth the results, generating the possible individual leaf models (Figure 1e–g). Leaf models with implausible shapes were then detected and rejected (Figure 1f,g). Two tests were applied to detect leaves with implausible shape. First, sharp changes in angle between adjacent segments along the path were detected. A leaf model was expected to have at most one large change in angle, where the leaf blade emerges from the leaf sheath. The number of sharp changes in angle was recorded for each leaf model. For the second test, we assumed that, due to gravity, the angle between segments of the leaf and the direction of gravity will decrease monotonically with distance from the highest point on the leaf. For each leaf, we recorded the number of segments which violate this assumption. For both tests, leaf models were rejected if the measured value was greater than the minimum value for any leaf model with the same base and end point. Filtering was again applied to remove very similar leaf models, removing models if the maximum deviation from another leaf model was <1.5 cm.

#### Plant reconstruction

A large set of possible plant models were generated with random selection from the candidate leaf models, and the best of these was selected as the final model. Models were evaluated based on the amount of plant structure covered in the image set, the number of pixels outside the plant which were covered, and the number of leaves used. The goal was to find a model which covered all of the visible plant using as few leaf models as possible, and with minimal coverage of image areas outside the plant. To determine coverage, the 2D path sets extracted for each image were divided into non‐overlapping segments, and the number of these segments intersected by the projection of a plant model into each view was counted.

The fit quality *Q*(*L*) for a given leaf set *L* is given by evaluating

$$Q\left(L\right)=|A_{L}\left|-\beta \right|B_{L}\left|-\gamma \right|C_{L}|$$

where,*A*_*L*_ is the set of segments intersected by a leaf point*B*_*L*_ is the set of leaf points not intersecting any segment*C*_*L*_ is the set of segments intersected by more than one leaf modelβ, γ are weights

Generation of an individual plant model *L* from a candidate set of leaf models *C* was performed according to the following algorithm:

**Table:** **Algorithm** Plant model generation

**while** |*C*| > 0 **do**
Remove a randomly selected leaf **L** from *C*
Set *T = L + * **{L}**
**if ** *Q*(*T*)* > Q*(*L*) **then**
Set *L = T*
Remove all leaves with the same end point as **L** from *C*
**end if**
**end while**

The top 1% of models found after 100 000 runs of this generation algorithm were retained, and considered a likely set of good plant reconstructions. Finally, from this set, we selected the model with the minimum total leaf curvature, to avoid selecting a model with unlikely deviations in leaf shape. The number of runs used was experimentally determined to be sufficient to find a model matching the plant. Similarly, other parameter values were chosen to give accurate results without requiring excessive processing time. The values can be reused for subsequent experiments in similar imaging conditions, without requiring adjustment. In its current form, the image classification and processing to generate the 100 000 candidate models takes approximately 2 min with an Intel^®^ Core™ i7‐7700 CPU with 3.60 GHz and 32 GB RAM. Our focus was on reducing the imaging time to eliminate the influence of circadian rhythm on plant images and a 2‐minute processing time would allow analysis of all images on a daily basis for an experiment of the size presented here and processing can be achieved with a commodity desktop computer.

#### Tracking leaf growth

A method of matching leaves between reconstructions of the same plant on different days was then applied, allowing for identification of the first leaf, second leaf, etc. This method compared leaves based on position, length, shape, and growth properties, and found an optimal assignment of leaf labels over time. For each leaf **L** on each date *n*, each possible assignment of a label for the subsequent date was evaluated to assign the following cost:

$$M\left(L_{n},{L_{n}}_{+1}\right)=A\left(L_{n},{L_{n}}_{+1}\right)+\beta B\left(L_{n},{L_{n}}_{+1}\right)+\gamma C\left(L_{n},{L_{n}}_{+1}\right)+\delta D\left(L_{n},{L_{n}}_{+1}\right)$$

where,*A*(**L**_*n*_,**L**_*n*+1_) is the average distance between a point on **L**_*n*_ and the nearest point on **L**_*n*+1_, divided by the maximum of the two leaf lengths. This penalises the overall shape change between the two leaves.*B*(**L**_*n*_,**L**_*n*+1_) is the difference between the angle between the up vector and a vector between the highest point on the leaf and the tip of the leaf, evaluated for both leaves. This penalises unlikely label assignments where a drooping leaf becomes upright on the subsequent date.*C*(**L**_*n*_,**L**_*n*+1_) measures any length increase for leaves where no growth has been seen on at least two consecutive dates, to penalise unlikely assignments where a leaf which has stopped growing resumes growth.*D*(**L**_*n*_,**L**_*n*+1_) is the difference between the length of leaf **L**_*n*+1_ and the predicted length of leaf **L**_*n*_ on date *n+*1, predicted from the length difference between date *n* and *n−*1.β, γ, δ are weights.

For each subsequent date, we selected the assignment which minimises the total cost. If two leaves emerged from one imaging date to the next, numbering was assigned randomly, apart from leaf 4 and emerging leaf (EL) 5, since leaf 4 was consistently longer. As it is possible that a leaf visible on one date will not be visible on the next date due to occlusion, we also allowed the label for a leaf to skip one or more dates, adding a penalty value *E* to the total cost if a label was skipped (Figure 1h–n).

#### Limitations

This method was specifically designed for reconstructing plant types with thin leaves. For such plants, we have found that the type of model used in this method, a set of 3D splines, provides an approximation of the plant structure that can be reliably extracted from an image set, and used to make accurate measurements of plant properties. This method will not be suitable for plants with broad leaves, where the greater image distance between points on the edge of a leaf and the leaf axis makes it more challenging to identify and reconstruct leaf axes, and to determine which leaf each plant pixel belongs to. However, a modified version of this generate‐and‐test method could be applied to such plants if a more appropriate method was used for reconstructing individual leaves, such as using feature matching to reconstruct points on the leaves, then fitting 3D surfaces through these points to model the leaves.

For accurate reconstruction of a leaf, this method requires that the majority of the length of the leaf is distinct and visible in at least two images. As such, it may not reconstruct very young leaves which cannot yet be easily distinguished from other plant structures. Similarly, accuracy will decline for more mature plants, where the greater structure density makes it harder to visually distinguish individual leaves. This method is intended for reconstructing plants in the early growth stages, and in our experiments, we have seen accurate reconstruction of plants with up to 14 leaves.

To identify leaves over time, the tracking component of this method assumes that leaves will have a similar appearance between image sets. As such, leaves may not be accurately identified if there is a large time gap between image sets being captured for the same plant. Ideally, new images will be captured every day.

### Phenotypic analysis

Initially for each plant, raw phenotypic measurements across days were smoothed using cubic smoothing splines. This approach does not make any assumptions about the shape of the temporal trend and has been found to be useful in similar experiments (Al‐Tamimi *et al*., 2016). The smoothed version of each of the phenotypic measurements was then used to estimate various growth‐related traits of the plants on a specific day as well as estimate RGR and AGR traits over specific time intervals between days. The smoothing and trait computations were achieved using imageData (Brien, 2017b) a package for the R statistical computing environment (R Core Team, 2017). These derived traits maintain data at the individual plant level and are subsequently used as response variables in the phenotypic and QTL analysis. In total, there were 84 responses investigated covering different traits and time intervals after salt inoculation.

Each response was analyzed using a baseline linear mixed model that accounted for spatial variation present in the Smarthouse (Brien *et al*., 2013) as well as appropriately modelled variation induced by the factors required for the design of the experiment. The maximal baseline model was

(1) y=Xβ+Zu+e

where **y** is the response vector of values for the trait being analyzed and ordered to ensure the control treatment was followed by the salt treatment, **β** is the vector of fixed effects, **u** is the vector of random effects with the design matrices **X** and **Z** respectively and **e** is the vector of residual effects**.**

The fixed effect vector, **β** ^⊤^ is partitioned as follows:

$$\left[\begin{array}{cccccccccc} \mu & P_{1} & P_{2} & P_{3} & P_{4} & \beta_{\mathrm{zone}}^{⊤} & \beta_{\mathrm{posnpair}}^{⊤} & \beta_{\mathrm{type}}^{⊤} & \beta_{\mathrm{treat}}^{⊤} & \beta_{\mathrm{int}}^{⊤} \end{array}\right]$$

where μ is the overall mean, *P*_j_, is a vector of coefficients accounting for additional structure in the population, **β**_zone_ and **β**_posnpair_ are vectors of the linear coefficients for trend within the Smarthouse associated with zone and pairs of positions corresponding to the main plots of the design, respectively, **β**_type_ is a vector with three parameters that correspond to the recombinant inbred (RI) lines and the parents Mundah and Keel, respectively, **β**_treat_ is a vector with a parameter for each for salt and control, and **β**_int_ is a vector with a parameter for each type‐treatment combination.

The random effects vector, **u** ^⊤^, is partitioned as

$$\left[\begin{array}{ccccccc} u_{\mathrm{szone}}^{⊤} & u_{\mathrm{sposnpair}}^{⊤} & u_{\mathrm{dzone}}^{⊤} & u_{\mathrm{dposnpair}}^{⊤} & u_{\mathrm{main}}^{⊤} & g_{C}^{⊤} & g_{S}^{⊤} \end{array}\right]$$

where **u**_*i*_ is the subvector of effects for the *i*th term, which has variance of the form $\theta_{i}^{2}I$. The subvectors are the coefficients of the spline basis functions for fitting smooth trends within the Smarthouse over zones and over the east‐west pairs of positions, deviations from these smooth trends, and main‐plot random effects within each zone, respectively. The random effects **g**_C_ and **g**_S_ are the vectors of effects for the RI lines for the control and salt treatment, respectively, having variance of the form $\theta_{\mathrm{gC}}^{2}I$ and $\theta_{\mathrm{gS}}^{2}I$, respectively. The variance for the residual term **e** is a block diagonal matrix with two blocks corresponding to the control and salt treatments, respectively, the blocks being of the form $\theta_{\mathrm{rC}}^{2}I$ and $\theta_{\mathrm{rS}}^{2}I$.

### Testing of model terms

For each of the maximal baseline models, residual maximum likelihood estimation (REML) ratio tests were used to test the inclusion of random terms corresponding to smooth trends and deviations from smooth trends over zones in the Smarthouse. Non‐significant terms were removed from the model. Additionally, the principal components (PCs) were tested for significance and removed from the model if their Wald statistic had a *P*‐value >0.05.

### Heritability, correlation & computations

The generalized heritability for each response was calculated using the formula derived in Cullis *et al*. (Cullis *et al*., 2006), namely $h^{2}=1-a/2\theta_{g}^{2}$ where *a* is the average pairwise prediction error variance of cultivar effects for a treatment (control or salt) and $\theta_{g}^{2}$ is the genetic variance of the treatment. For each of the traits exhibiting heritability greater than 0.1, the Best Linear Unbiased Predictors (BLUPs) of the lines for each treatment were then extracted from the linear mixed model of the phenotypic analysis. Independently for each treatment, estimated genetic correlations were calculated for every pairwise combination of trait BLUPs (Tables S6 and S7, Figure S6) using the rcorr function of the R library Hmisc (Harrell, 2018). The flexible linear mixed model package ASReml‐R (Butler *et al*., 2009) was used for all phenotypic modelling and testing of model terms was conducted using ASRemlPlus (Brien, 2017a), both of which are R‐packages.

### Genotyping

Genomic DNA was extracted from young leaves using the phenol/chloroform method (Rogowsky *et al*., 1991). DNA concentration and quality were analyzed using a NanoDrop 1000 Spectrophotometer (Thermo Fisher Scientific, Wilmington, DE, USA) and quantified using the PicoGreen method (Ahn *et al*., 1996). DNA concentrations were then normalized to 20 ng μl^−1^. The population was genotyped using genotyping‐by‐sequencing (GBS) with libraries prepared according to the methods described by Elshire *et al*. (Elshire *et al*., 2011) and Poland *et al*. (Poland *et al*., 2012). Each DNA sample was digested using two restriction enzymes, *Pst*I and *Msp*I, for complexity reduction and was then barcoded with a unique variable length DNA sequence adapter. Samples were multiplexed with each library containing 95 DNA samples and a water control which was run on a single lane of an Illumina HiSeq2000. The Illumina HiSeq raw sequencing files were initially subjected to a quality control (QC) assessment using the FastQC quality checking software available at http://www.bioinformatics.babraham.ac.uk/projects/fastqc. SNP calling was performed using the non‐reference genome Universal Network‐Enabled Analysis Kit (UNEAK) protocol (Lu *et al*., 2013) implemented in TASSEL v3.0.158 (Bradbury *et al*., 2007; Glaubitz *et al*., 2014). Modules in TASSEL‐GBS pipeline were executed sequentially, and reporting logs were manually screened for errors using custom bash scripts. Reads were trimmed to 64 bases with a minimum tag count of five. To identify pairs of tags for SNP calling, the error tolerance rate was set to 0.03. Only SNPs with a minor allele frequency of 0.05 were used for further analyses.

### Linkage map construction and population structure analysis

Linkage map was constructed using R‐packages R/qtl (Broman and Sen, 2009) and R/ASMap (Taylor and Butler, 2017) available at R (R Core Team, 2017). The initial TASSEL output identified 28,810 SNP markers which were assessed for suitability. Of these 3,784 markers which had <20% missing data were taken forward for genetic map construction. From this subset of markers, lines with more than 50% missing data and 20% missing allele scores were discarded, and genetic similarity between lines and sibling relatedness between individuals was estimated. ‘Partial clone’ groups were formed by members with 85% allelic similarity, and one member from each group with the least missing values was used as representative for map construction. Marker segregation distortion was calculated using a Bonferroni corrected *P*‐value of 0.05 to remove groups with higher distortion. The remaining markers were then clustered into linkage groups and optimally ordered using MSTmap (Wu *et al*., 2008) available at ASMap. Recombination rates were calculated and markers above the median recombination rate for the population, using Bonferroni corrected *P *<* *0.05, were removed. Markers within linkage groups were re‐ordered and marker/interval statistics calculated. Markers containing significantly greater (Bonferroni corrected *P *< 0.05) double recombinations in comparison to the recombinations in adjacent intervals were removed. Finally, markers within linkage groups were optimally ordered and genetic distances calculated. Linkage group identification and orientation were determined by aligning marker sequences to the barley reference sequence assembly (Mayer *et al*., 2012) through an in‐house BLAST portal. The final linkage map consisted of 298 lines genotyped with 3,087 GBS markers and 10 linkage groups. Where two markers had a distance >30 cM, the linkage group was split in two. Total map length was 1303.86 cM with an average interval spacing of 0.42 cM.

After linkage map construction, a PC analysis using the R library lmem.gwaser (Gutierrez *et al*., 2016) was conducted in order to investigate remaining individual RIL lines with high similarity. There was evidence of tight coupling between some individuals in plots of the PCs suggesting that some residual relationships were still present in the data set. As this population structure still present within the data may affect the level of false positives in the QTL mapping, we included the first four PCs which accounted for around 28% of the genetic variation in the data in the phenotypic analysis.

### QTL mapping

A WGAIM approach was used for QTL analysis of the response variables (Verbyla *et al*., 2007, 2012), using the R package WGAIM (Taylor and Verbyla, 2011), with independent analyses conducted for the control and salt treatments. Analyses were only conducted if the response variable showed sufficient genetic variation evidenced by a heritability greater than 0.1. The WGAIM approach uses the complete phenotypic information for both treatments, control and salt, and ensures that parameters such as the design factors, spatial variation and population structure PCs are simultaneously estimated with marker effects; thus increasing the power of detecting significant interval marker trait associations (Verbyla *et al*., 2007). To detect and select significant interval markers linked to the trait, WGAIM uses a forward selection approach. This approach avoids piecemeal scanning of individual markers and calculation of thresholds by directly testing the significance of the additive genetic variance parameter using a REML ratio test. If significant, an outlier detection method is then used to select the most likely interval marker linked to a putative QTL. This interval marker is removed from the contiguous block and placed as a separate term in the fixed component of the extended model. This procedure is then repeated until the genetic variance parameter is non‐significant. This algorithm has been shown to detect and select putative QTL with a Type I family‐wise error rates below 5% and stable false discovery rates for various population and marker set sizes (Verbyla *et al*., 2007). Upon completion of the algorithm, all significant QTL are summarized and reported. This summary includes selected interval markers considered to be significant with their respective flanking markers, size of the interval marker effect, *P*‐value based on *Z*‐statistic (α < 0.05), and LOD scores for each QTL.

### Candidate gene analysis

Candidate gene was identified for all the significant QTL intervals (interval between flanking markers), using the reference sequence of the barley genome (Mascher *et al*., 2017) as deposited on the IPK Barley BLAST Server (http://webblast.ipk-gatersleben.de/barley_ibsc/). Custom Linux script was used to perform a BLAST search of the GBS marker's sequence against the pseudomolecule sequences for each individual chromosome. After the BLAST search, two possible scenarios occurred: (i) if a hit was obtained for both flanking markers, then the interval was defined as the physical coordinates between each flanking marker; and (ii) if a hit was found for only one of the flanking markers, then an interval of 500 kb surrounding the flanking maker was used to identify the annotated genes present in the mapping interval using BEDTools (Quinlan and Hall, 2010).

### Data and code availability

3D modelling – code is available upon request from Anton van den Hengel R‐packages used in this study have been published and are available online.

## Author Contributions

B.W. developed the structural model approach and analyzed the imaging data. R.S., S.R. and B.B supervised the phenotypic experiments. C.B. designed the experiments and performed the growth analysis. A.P., A.T. and J.T. genotyped the population and constructed the genetic map. H.O. performed the QTL mapping. S.N. and D. J. performed the genetic analysis of QTL regions. M.T and A.v.d.H. conceived the study. A.v.d.H. supervised the modelling work. All authors contributed to writing and reviewing the manuscript.

## Conflict of Interest

The authors declare no competing financial interests.

## Supporting information


**Figure S1.** Images of example camera calibration targets and 3D leaf segments prior to assembly of 3D image path.
**Figure S2.** Correlation between manual leaf length measurements and digital measurement of leaf length in *Hordeum vulgare* and *Triticum aestivum* plants of different growth stages.
**Figure S3.** Overlay of 3D leaf model of *Hordeum vulgare* with original images used to create the model and 3D model projected to unused top view image.
**Figure S4.** Flowchart of data processing, from image capture to QTL analysis, including software packages used.
**Figure S5.** Confusion matrix for the binary SVM classifier, tested on five manually labelled images.
**Figure S6.** Genetic correlation between traits, based on the correlation between the Best Linear Unbiased Predictors (BLUPs) for control and salt traits.

**Table S1.** Trait description – Description of all 2D and 3D traits extracted from the image analysis and time intervals analysed for the *Hordeum vulgare* mapping population.
**Table S2.** Heritability – Values of heritability determined for individual traits listed in Table S1, traits with h > 0.1 were used for QTL analysis of the *Hordeum vulgare* mapping population.
**Table S3.** Significant QTL – overview of all significant QTL with time intervals determined by wgaim method for the *Hordeum vulgare* mapping population.
**Table S4.** Overview QTL – Table of all significant QTL sorted by 2D and 3D traits and chromosome position for the *Hordeum vulgare* mapping population.
**Table S5.** Candidate genes – List of all candidate genes identified between the flanking markers for the respective QTL based on the latest release of the *Hordeum vulgare* genome.
**Table S6.** Genetic correlation between traits, based on the correlation between the Best Linear Unbiased Predictors (BLUPs) – control traits.
**Table S7.** Genetic correlation between traits, based on the correlation between the Best Linear Unbiased Predictors (BLUPs) – salt traits.
