# Supplementary material for: High‐throughput 3D modelling to dissect the genetic control of leaf elongation in barley (Hordeum vulgare)
Source: Plant J. 2019 Feb 22;98(3):555–70. doi: 10.1111/tpj.14225 (PMC6850118; doi:10.1111/tpj.14225)
Supplement: Supplementary file 1 — Figure S1. Images of example camera calibration targets and 3D leaf segments prior to assembly of 3D image path. Figure S2. Correlation between manual leaf length measurements and digital measurement of leaf length in Hordeum vulgare and Triticum aestivum plants of different growth stages. Figure S3. Overlay of 3D leaf model of Hordeum vulgare with original images used to create the model and 3D model projected to unused top view image. Figure S4. Flowchart of data processing, from image capture to QTL analysis, including software packages used. Figure S5. Confusion matrix for the binary SVM classifier, tested on five manually labelled images. Figure S6. Genetic correlation between traits, based on the correlation between the Best Linear Unbiased Predictors (BLUPs) for control and salt traits. [file TPJ-98-555-s001.pdf]

SUPPORTING FIGURES

Ward et al. - High-throughput 3D modelling to dissect the genetic control of leaf elongation in barley (*Hordeum vulgare*)

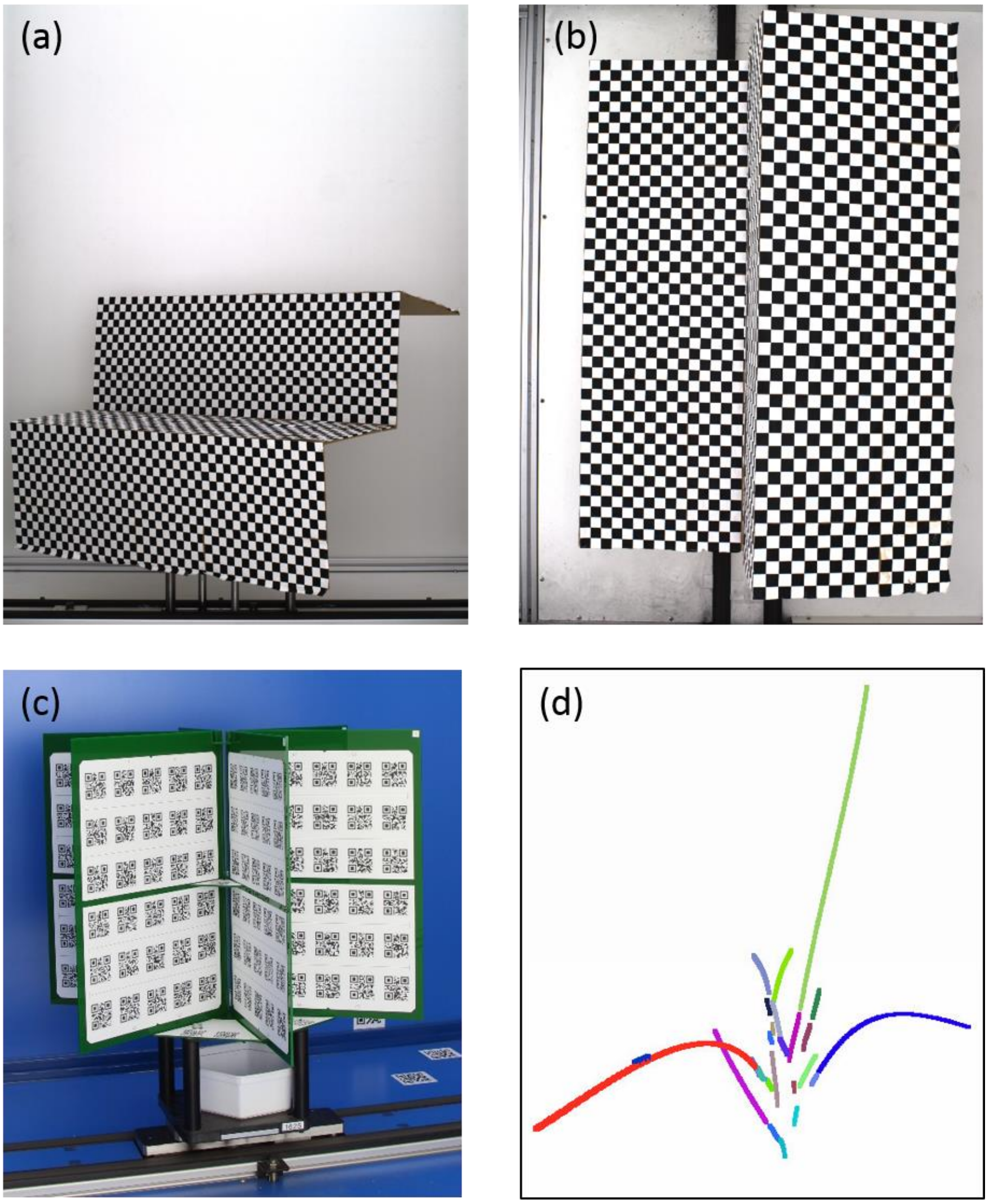

Figure S1. Images of example camera calibration targets and 3D leaf segments prior to assembly of 3D image path. (a) Side view and (b) top view photograph of calibration object with chess board pattern used to determine camera position and orientation. (c) Example of possible calibration target for routine usage on conveyor-based imaging system; (d) Example of individual 3D leaf segments identified prior to assembly of 3D leaf paths.

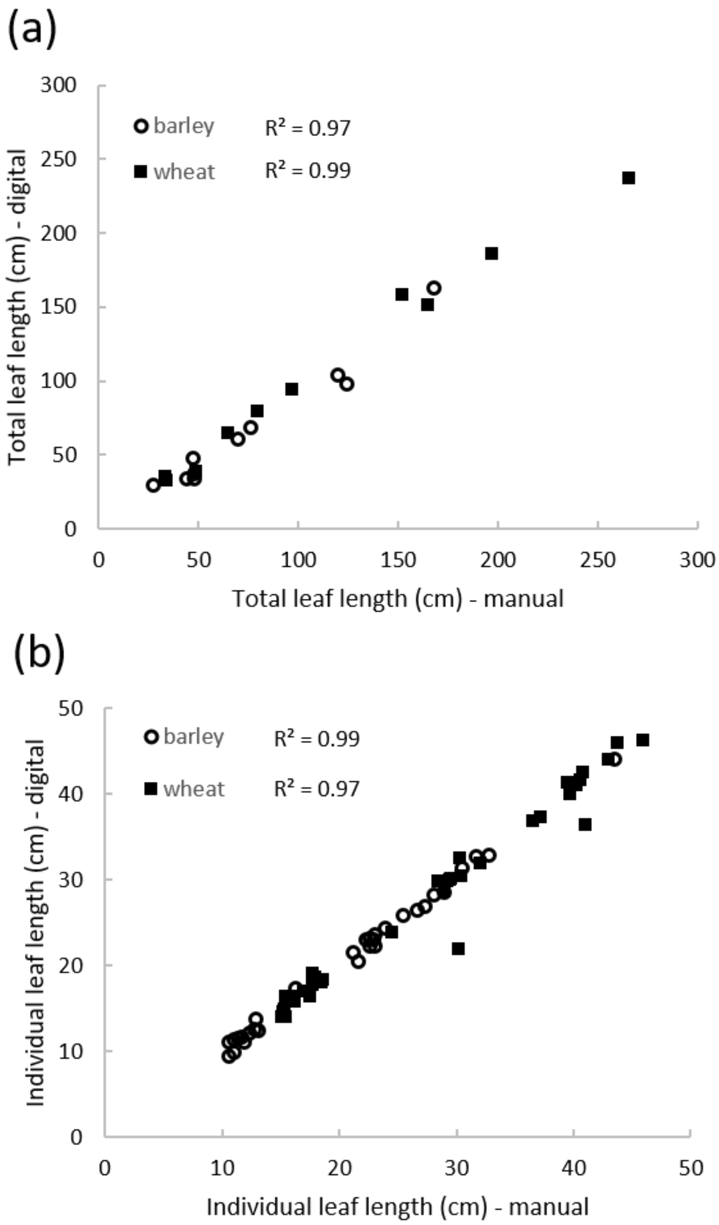

Figure S2. Correlation between manual leaf length measurements and digital measurement of leaf length in *Hordeum vulgare* and *Triticum aestivum* plants of different growth stages. Correlation between manual and digital measurements of (a) total leaf length and (b) individual leaf lengths of ten wheat and ten barley plants. R^2: squared linear correlation coefficients.

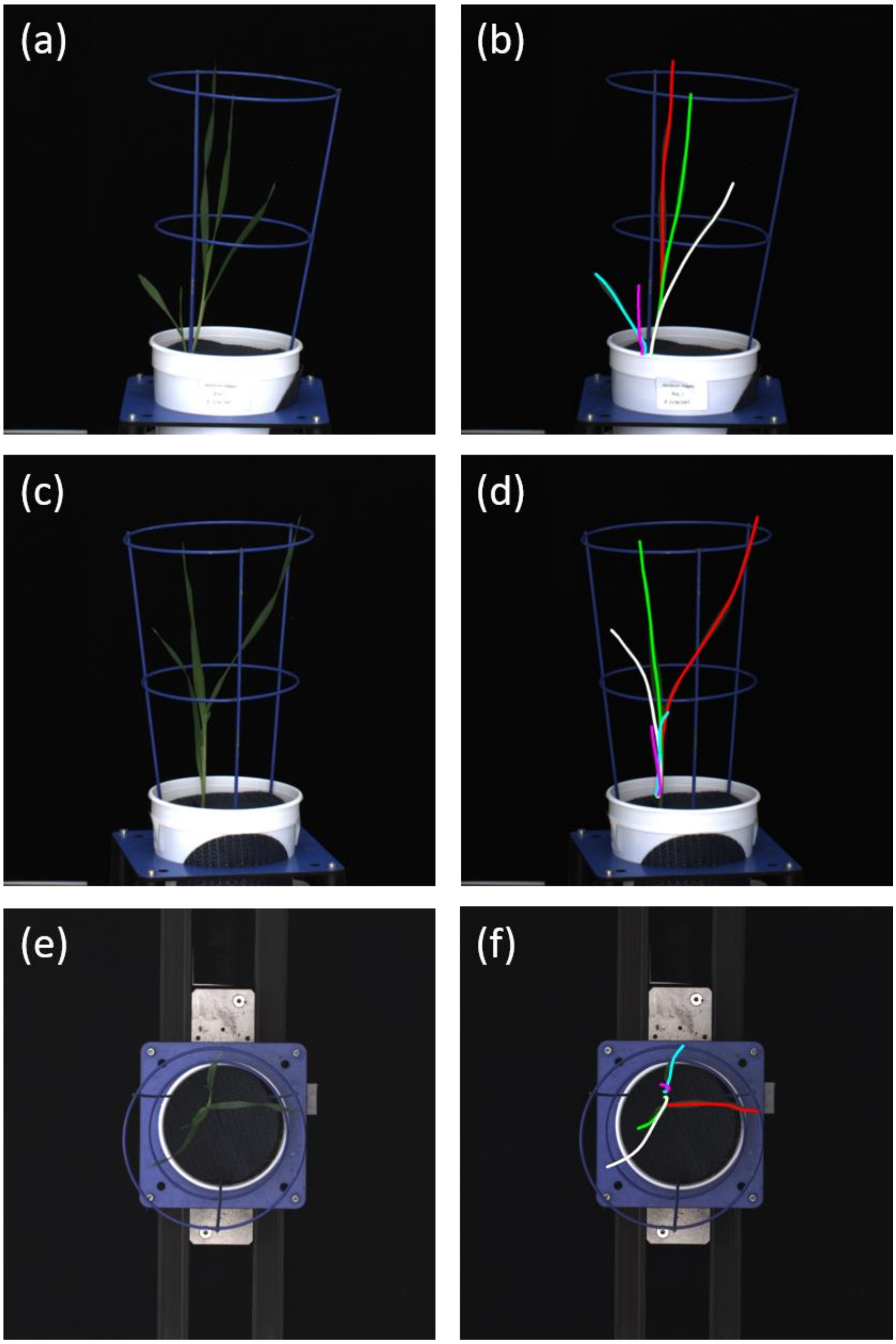

Figure S3. Overlay of 3D leaf model of *Hordeum vulgare* with original images used to create the model and 3D model projected to unused top view image. (a, c) Original RGB side view images of an example barley plant at different rotational angels with (b, d) 3D leaf model projected onto RGB images. Both images were used for 3D reconstruction. (e) RGB top view image of same plant with (f) overlay of 3D model onto top view image. This image was not used for the 3D modelling.

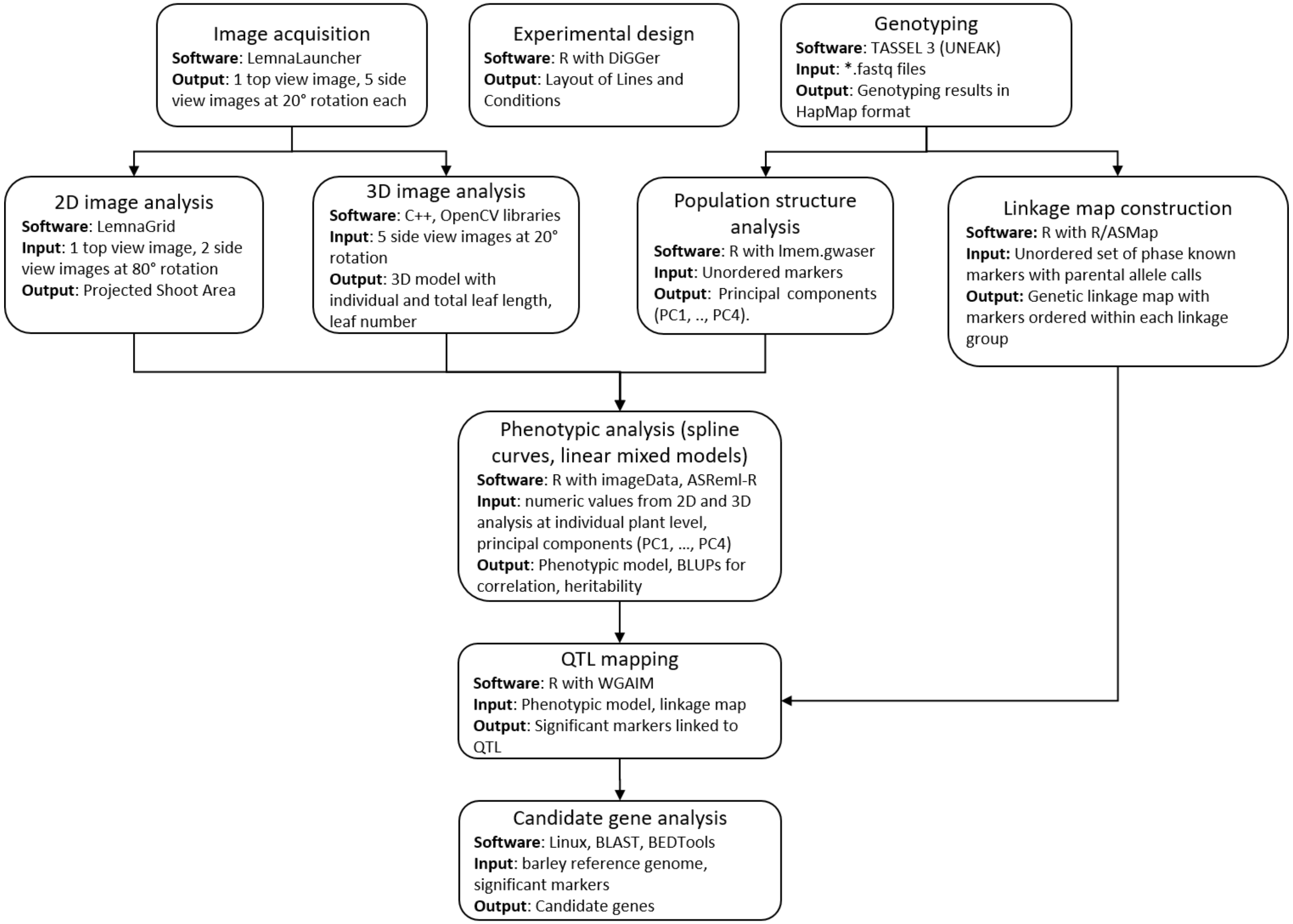

Figure S4. Flowchart of data processing steps from raw data acquisition to candidate gene analysis. Software tools used are listed, including R packages.

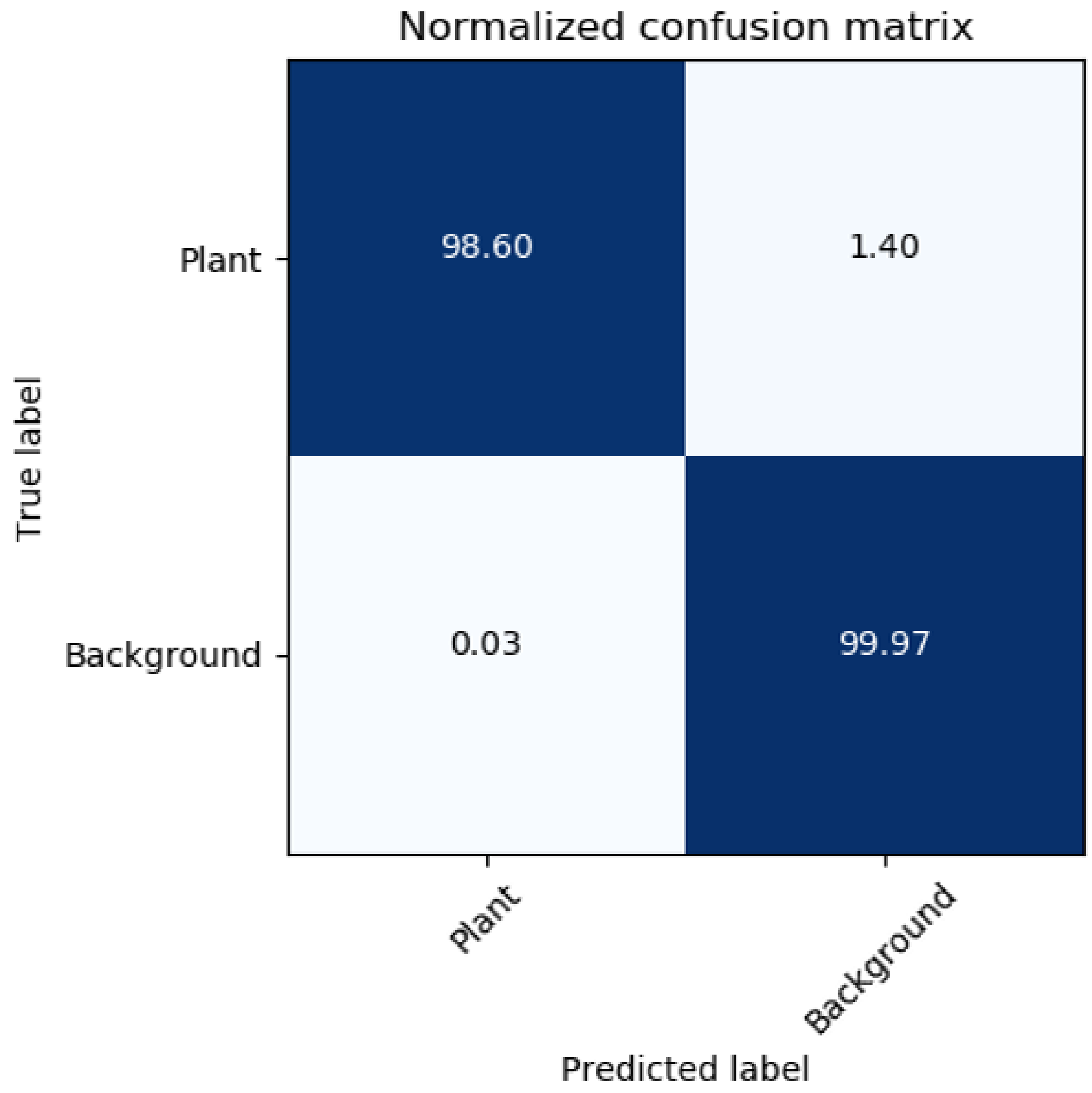

Figure S5. Confusion matrix for the binary SVM classifier, tested on five manually labelled images.

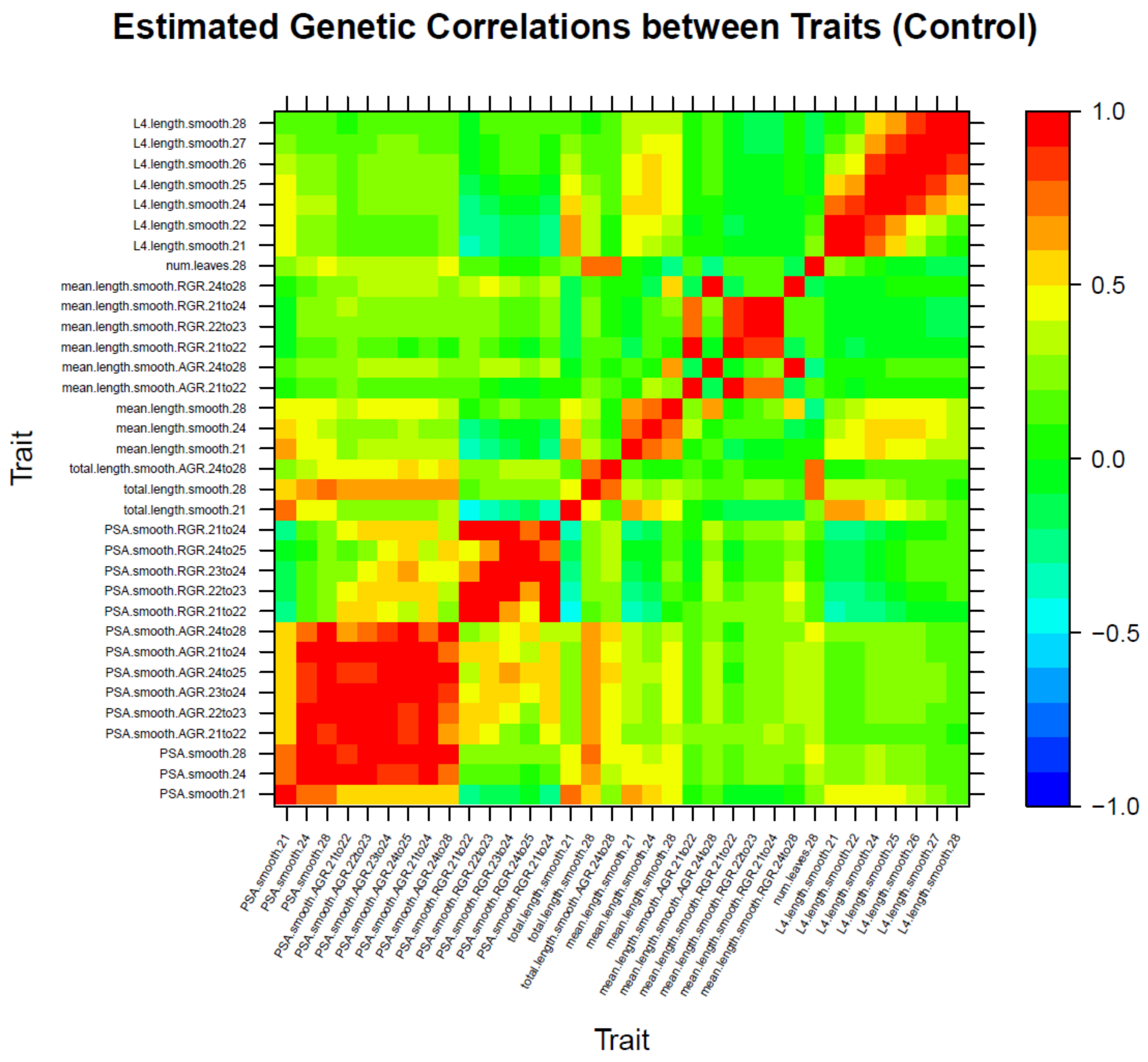
Estimated Genetic Correlations between Traits (Control)
Trait
L4.length.smooth.28
L4.length.smooth.27
L4.length.smooth.26
L4.length.smooth.25
L4.length.smooth.24
L4.length.smooth.22
L4.length.smooth.21
num.leaves.28
mean.length.smooth.RGR.24to28
mean.length.smooth.RGR.21to24
mean.length.smooth.RGR.22to23
mean.length.smooth.RGR.21to22
mean.length.smooth.AGR.24to28
mean.length.smooth.AGR.21to22
mean.length.smooth.28
mean.length.smooth.24
mean.length.smooth.21
total.length.smooth.AGR.24to28
total.length.smooth.28
total.length.smooth.21
PSA.smooth.RGR.21to24
PSA.smooth.RGR.24to25
PSA.smooth.RGR.23to24
PSA.smooth.RGR.22to23
PSA.smooth.RGR.21to22
PSA.smooth.AGR.24to28
PSA.smooth.AGR.21to24
PSA.smooth.AGR.24to25
PSA.smooth.AGR.23to24
PSA.smooth.AGR.22to23
PSA.smooth.AGR.21to22
PSA.smooth.28
PSA.smooth.24
PSA.smooth.21
1.0
0.5
0.0
−0.5
−1.0
Trait

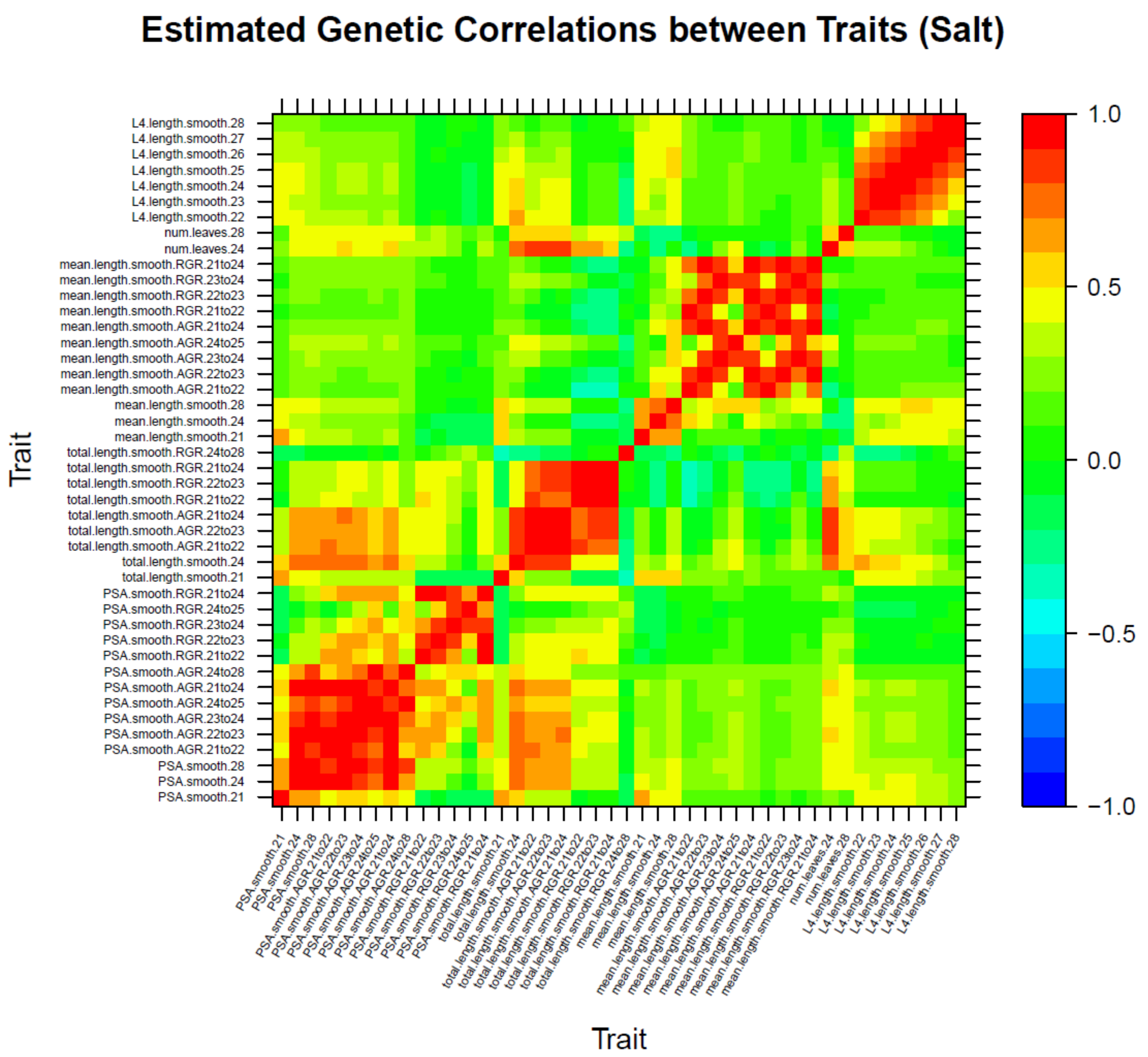
Estimated Genetic Correlations between Traits (Salt)
Trait
L4.length.smooth.28
L4.length.smooth.27
L4.length.smooth.26
L4.length.smooth.25
L4.length.smooth.24
L4.length.smooth.23
L4.length.smooth.22
num.leaves.28
num.leaves.24
mean.length.smooth.RGR.21to24
mean.length.smooth.RGR.23to24
mean.length.smooth.RGR.22to23
mean.length.smooth.RGR.21to22
mean.length.smooth.AGR.21to24
mean.length.smooth.AGR.24to25
mean.length.smooth.AGR.23to24
mean.length.smooth.AGR.22to23
mean.length.smooth.AGR.21to22
mean.length.smooth.28
mean.length.smooth.24
mean.length.smooth.21
total.length.smooth.RGR.24to28
total.length.smooth.RGR.21to24
total.length.smooth.RGR.22to23
total.length.smooth.RGR.21to22
total.length.smooth.AGR.21to24
total.length.smooth.AGR.22to23
total.length.smooth.AGR.21to22
total.length.smooth.24
total.length.smooth.21
PSA.smooth.RGR.21to24
PSA.smooth.RGR.24to25
PSA.smooth.RGR.23to24
PSA.smooth.RGR.22to23
PSA.smooth.RGR.21to22
PSA.smooth.AGR.24to28
PSA.smooth.AGR.21to24
PSA.smooth.AGR.24to25
PSA.smooth.AGR.23to24
PSA.smooth.AGR.22to23
PSA.smooth.AGR.21to22
PSA.smooth.28
PSA.smooth.24
PSA.smooth.21
Trait
1.0
0.5
0.0
−0.5
−1.0

Figure S6. The genetic correlation between traits based on the correlation between the Best Linear Unbiased Predictors (BLUPs) or predicted effects of each line for control and salt. The BLUPs are obtained from the results of the final phenotypic analysis for each trait. The correlation between traits with heritability greater than 0.1 for each treatment was calculated separately on pairs of trait data from the same line.
