# Supplementary material for: High‐throughput 3D modelling to dissect the genetic control of leaf elongation in barley (Hordeum vulgare)
Source: Plant J. 2019 Feb 22;98(3):555–70. doi: 10.1111/tpj.14225 (PMC6850118; doi:10.1111/tpj.14225)
Supplement: Supplementary file 3 [file TPJ-98-555-s003.docx]

**SHORT SUPPORTING LEGENDS**

**Figure S1.** Images of example camera calibration targets and 3D leaf segments prior to assembly of 3D image path.

**Figure S2.** Correlation between manual leaf length measurements and digital measurement of leaf length in *Hordeum vulgare* and *Triticum aestivum* plants of different growth stages.

**Figure S3.** Overlay of 3D leaf model of *Hordeum vulgare* with original images used to create the model and 3D model projected to unused top view image.

**Figure S4.** Flowchart of data processing, from image capture to QTL analysis, including software packages used.

**Figure S5.** Confusion matrix for the binary SVM classifier, tested on five manually labelled images.

**Figure S6.** Genetic correlation between traits, based on the correlation between the Best Linear Unbiased Predictors (BLUPs) for control and salt traits.

**Table S1.** Trait description - Description of all 2D and 3D traits extracted from the image analysis and time intervals analysed for the *Hordeum vulgare* mapping population.

**Table S2.** Heritability - Values of heritability determined for individual traits listed in Table S1, traits with h>0.1 were used for QTL analysis of the *Hordeum vulgare* mapping population.

**Table S3.** Significant QTL - overview of all significant QTL with time intervals determined by wgaim method for the *Hordeum vulgare* mapping population.

**Table S4.** Overview QTL - Table of all significant QTL sorted by 2D and 3D traits and chromosome position for the *Hordeum vulgare* mapping population.

**Table S5.** Candidate genes - List of all candidate genes identified between the flanking markers for the respective QTL based on the latest release of the *Hordeum vulgare* genome.

**Table S6.** Genetic correlation between traits, based on the correlation between the Best Linear Unbiased Predictors (BLUPs) - control traits.

**Table S7.** Genetic correlation between traits, based on the correlation between the Best Linear Unbiased Predictors (BLUPs) - salt traits.
